# Sex-specific effects of a high fat diet on aortic inflammation and dysfunction

**DOI:** 10.1038/s41598-023-47903-1

**Published:** 2023-12-08

**Authors:** Vivian Tran, Holly Brettle, Henry Diep, Quynh Nhu Dinh, Maeve O’Keeffe, Kerry V. Fanson, Christopher G. Sobey, Kyungjoon Lim, Grant R. Drummond, Antony Vinh, Maria Jelinic

**Affiliations:** 1 Centre for Cardiovascular Biology and Disease Research, Department of Microbiology, Anatomy Physiology and Pharmacology, School of Agriculture, Biomedicine and Environment, La Trobe University, Bundoora, VIC Australia; 2 Department of Animal, Plant and Soil Sciences, School of Agriculture, Biomedicine and Environment, La Trobe University, Bundoora, VIC Australia

**Keywords:** Aortic diseases, Obesity

## Abstract

Obesity and vascular dysfunction are independent and sexually dimorphic risk factors for cardiovascular disease. A high fat diet (HFD) is often used to model obesity in mice, but the sex-specific effects of this diet on aortic inflammation and function are unclear. Therefore, we characterized the aortic immune cell profile and function in 6-week-old male and female C57BL/6 mice fed a normal chow diet (NCD) or HFD for 10 weeks. Metabolic parameters were measured weekly and fortnightly. At end point, aortic immune cell populations and endothelial function were characterized using flow cytometry and wire myography. HFD-male mice had higher bodyweight, blood cholesterol, fasting blood glucose and plasma insulin levels than NCD mice (*P* < 0.05). HFD did not alter systolic blood pressure (SBP), glycated hemoglobin or blood triglycerides in either sex. HFD-females had delayed increases in bodyweight with a transient increase in fasting blood glucose at week 8 (*P* < 0.05). Flow cytometry revealed fewer proinflammatory aortic monocytes in females fed a HFD compared to NCD. HFD did not affect aortic leukocyte populations in males. Conversely, HFD impaired endothelium-dependent vasorelaxation, but only in males. Overall, this highlights biological sex as a key factor determining vascular disease severity in HFD-fed mice.

## Introduction

Obesity is a complex and multifaceted condition that affects approximately 11% men and 15% women worldwide^1,2^. It is characterized by an excessive accumulation of fat and increased risk of metabolic disorders such as type-2 diabetes mellitus, fatty liver disease, Alzheimer's disease, and cardiovascular diseases such as hypertension, myocardial infarction and stroke^3,4^. Due to the consumption of caloric dense foods, overconsumption of calories is integral to an “unhealthy” diet. As a result, increased fat consumption has been considered responsible for obesity and its associated metabolic diseases^5^. Despite various prevention and treatment strategies, including lifestyle changes, drug therapies and surgery, obesity remains challenging to manage, likely due to its multifactorial nature^6^. The global prevalence of overweight and obesity is higher in women than in men, with striking sexual dimorphisms in the metabolic disturbances that occur in the later stages of obesity^7^. While these sex-specific differences are well-reported, little is known regarding any differences that may be present in the early stages of obesity.

The impact of biological sex is often overlooked in biomedical research with many preclinical studies focusing on males, which has substantially hindered the relevance of findings to half of the population^8^. To better understand the progression of obesity-related disorders, monogenetic, polygenetic, surgical and diet-induced rodent models have been used^9^. Diet-induced rodent models comprising dietary imbalances (i.e. excessive caloric intake) are widely used to model clinical obesity^10^, and commonly used are high-fat diets (HFDs) containing ~ 40–60% of energy derived from lipids and fats to increase caloric intake^11^. As in clinical obesity, sex differences (such as adipose tissue distribution and energy expenditure) are also present in HFD mouse models of obesity^12^. Typically, male mice develop severe obesity, along with metabolic complications such as hyperinsulinemia, hyperglycemia, and hypertension. Many studies exclude female mice as they are more resistant to HFD-induced metabolic complications^13–15^. The studies that do include both sexes all involve long diet regimens, which model the advanced or late stages of HFD-induced obesity. As a result, there is a paucity of research in the sex differences and sex-specific effects of in the early stages of HFD-induced obesity. Considering how commonly the HFD is used to model obesity in mice, this is an important area of research to address.

Aortic dysfunction and stiffening are major consequences of obesity and drivers of the subsequent end-organ damage. Endothelial dysfunction, in particular, is one of the earliest vascular alterations in obesity^16^. The mechanisms underlying endothelial dysfunction are largely attributed to reduced nitric oxide production and bioavailability^16^. However, there have been inconsistent reports on the effects of obesity on vascular function due to variations in diet composition and duration^17–20^, with most studies using males only.

To understand the correlation between obesity and cardiovascular risk, recent research has focused on understanding the changes that occur in fat depots in obesity, including perivascular adipose tissue. This is important as perivascular adipose tissue surrounds blood vessels and releases a wide variety of adipokines, allowing for cellular communication and changes in vascular function^21,22^. Under conditions such as obesity, perivascular adipose tissue will undergo hypertrophy and hyperplasia. However, it is also typically accompanied with a reduction in angiogenesis, which in turn causes hypoxia^23^. These hypoxic conditions stimulate the production of proinflammatory cytokines and the recruitment of immune cells into the perivascular adipose tissue^24^. The chronic low-grade inflammation can promote oxidative stress and the dysregulation of adipokines released from the perivascular adipose tissue. This concomitantly interferes with vascular function^21^.

Inflammation is a well-recognized driver of endothelial dysfunction and vascular stiffening. In the later stages of HFD-induced obesity, circulating macrophages, neutrophils and T cells are increased. This inflammatory response to a HFD is exacerbated in males compared to females^25^. Infiltration of immune cells into the aortic wall is a critical aspect of aortic disease in obesity, involving production of local proinflammatory cytokines to promote arterial stiffening and dysfunction^26,27^. However, there is still little information on any sex-related differences in aortic immune cells during a HFD. It is thus important to characterize the early local inflammatory response to HFD-induced obesity within the aorta, and any sex-specific effects in this process. Thus, this study aimed to characterise the metabolic, vascular and inflammatory responses to a 10-week HFD regimen in male and female C57BL/6 mice.

## Methods

### Animal model of obesity

All animal experimentation complied with the National Health and Medical Research Council (NHMRC) of Australia code of practice for the care and use of animals for scientific purposes and ARRIVE guidelines. Animal care and experimental protocols were approved by the La Trobe Animal Ethics committee (AEC19009). Male and female C57BL/6 J mice were obtained from the La Trobe Animal Research and Teaching Facility and housed in individually ventilated cages with access to food and water ad libitum. At 6 weeks of age, mice were randomly assigned to either a normal chow diet (NCD; 20% total crude protein, 5% crude fat, 6% crude fiber, 0.5% added salt, 0.8% calcium and 0.45% phosphorus; Barastoc WEHI mouse breeder cubes Irradiated; Ridley, Australia) or a semi-pure HFD (19.4% protein, 23% total fat, 4.66% crude fiber, 4.66% AD fiber; SF03-030, Specialty Feeds) for 10 weeks during which bodyweight was recorded weekly.

### Blood pressure measurement

Systolic BP (SBP) was measured weekly by a non-invasive tail cuff plethysmography (MC4000 Multi Channel Blood Pressure Analysis System, Hatteras Instruments, Cary, NC, USA) as previously described^28^. Mice were acclimated 1 week prior to BP measurements to minimize stress. Mice underwent 10 acclimation cycles, followed by 4 sets of 15 cycles. The data output was recorded using MC4000 Blood Pressure Analysis System software. Mean SBP measurements were recorded weekly during the 10-week HFD regimen.

### Fasted blood collection

Fasted bloods were collected fortnightly. Mice were fasted for 6 h (from 8:00 AM) with free access to water. Blood glucose was measured in samples from the saphenous vein using a handheld Accu-Chek® Guide glucose meter per manufacturer’s instructions (NSW, Australia). Blood was collected (~ 120μL) for plasma isolation to measure insulin using a Mouse Ultrasensitive Insulin ELISA kit (ALPCO Diagnostics, Salem, NH, USA) as per manufacturer’s instructions^29^.

### Plasma estradiol measurement

Plasma steroids were extracted twice with diethyl ether to remove potential interfering substances^30^. The supernatant, containing extracted steroids, was collected, dried and reconstituted in phosphate buffer. Plasma was analyzed using an oestradiol antibody (ISWE0008, Arbor Assays, Ann Arbor, Michigan). Briefly, a 96-well flat-bottomed microtiter plate was coated with *donkey anti*-*sheep immunoglobulin*. The plate was washed and samples, standards (10–0.078 ng/ml) and controls were loaded into each well, followed by the corresponding enzyme-labelled antibody. The plate was incubated for 2 h then washed. TMB substrate solution was added to each well. After 30 min, the reaction was stopped using 0.5 M H_2_SO_4_ and the plate was read at 450 nm and 620 nm using the SPECTROstar Nano Plate reader (BMG Labtech, Mornington Victoria, Australia). All samples were analysed in duplicate. To assess matrix interference and determine the dilution factor for each assay, a parallelism between the standard curve and a serially diluted sample pool was performed for all samples.

### Isolation of aortae

Mice were euthanized using carbon dioxide asphyxiation followed by cardiac puncture and injection of 0.1 mL of enoxaparin sodium (400 units; Clexane®, Auckland, NZ). Blood samples were measured for glycated hemoglobin (HbA1c), total cholesterol and triglycerides using the Roche Cobas b 101 Monitoring System (Basel, Switzerland^29^). Mice were intracardially perfused with phosphate-buffered saline (PBS). Aortae were harvested, then placed in ice-cold PBS for either flow cytometry, histology or molecular analyses. Lymph nodes and excess visceral fat were carefully removed via microdissection^31^. For wire myography studies, and after perivascular adipose tissue removal, aortae were dissected in ice-cold Krebs bicarbonate solution (mmol/L; NaCl 2973.1, KCl 117.4, MgSO_4_.7H_2_O 29.3, KH_2_PO_4_ 29.6).

### Flow cytometry

Flow cytometry was performed on cell suspensions derived from freshly isolated aortae (including perivascular adipose tissue) as previously described^31^. Aortae were minced with scissors and enzymatically digested in a buffer (MgCl_2_ and CaCl_2_) comprising a mixture of type XI collagenase (125 U/mL, C7657-100MG), hyaluronidase (600 U/mL, H3884-50MG) and type I-S collagenase (450 U/mL, C1639-50MG; all enzymes purchased from Sigma-Aldrich, USA) for 1 h at 37 °C, as previously described^31^. Samples were then passed through a sterile 70 μm filter (BD Biosciences, USA) and cells were pelleted by centrifugation at 1500 RPM for 5 min at 4 °C. Aortic cell suspensions were stained with an Aqua Live/dead cell viability stain (Invitrogen, USA; 1 in 1000 dilution in PBS) for 15 min at room temperature, followed by a master mix containing a cocktail of antibodies in MACS buffer (0.5% bovine serum albumin and 2 mM EDTA in PBS; full list of antibodies in Suppl Table 1). Lastly, stained cells were fixed in MACS buffer containing 1% formalin. All samples were analyzed using a CytoFLEX S analyser (Beckman Coulter, Indianapolis, IN, USA) using CytExpert software version 2.5 (Beckman Coulter, USA). Immune cell populations were quantified using FlowJo software (BD Biosciences, version 10.8.1; gating strategy in Suppl Fig. 1). Cell numbers were expressed as total cells per aorta.

### Wire myography

Vascular function of the abdominal aorta was assessed as described previously^32^. Aortic rings (2 mm in length) were mounted on a 4-channel Mulvany-Halpern Myograph (model 610 M, Danish Myo Technology, Aarhus, Denmark), and allowed to stabilize at zero tension for 15 min followed by normalization and equilibration^33^. All experiments were performed in Krebs bicarbonate solution at 37 °C and bubbled with carbogen (95% O_2_ and 5% CO_2_). Changes in isometric tension were recorded using a Powerlab/Lab Chart data acquisition system (AD Instruments, Bella Vista, NSW, Australia). Vessels were maximally contracted with the thromboxane A_2_ mimetic U46619 (1 μM bolus). This was followed by the determination of endothelial integrity as described previously^34,35^. To assess vasorelaxant function, aortic rings were pre-contracted to 70–80% of maximum U46619 contraction, using U46619, and cumulative concentration–response curves to the endothelium-dependent agonist acetylcholine (ACh; 0.1 nM–10 μM) or the endothelium-independent vasorelaxant NO donor sodium nitroprusside (SNP; 0.1 nM–10 μM) were performed, as previously described^33^. Additionally, responses to ACh and U46619 were examined after a 30 min incubation in the presence of a NO synthase inhibitor, Nω-nitro-ι-arginine methyl ester (L-NAME; 200 μM) or indomethacin (1 μM), a cyclooxygenase (COX) inhibitor. To assess this response, aortic rings were then pre-contracted to 50–70% of maximum U46619 contraction using U46619, and cumulative concentration–response curves to endothelial agonist acetylcholine (ACh; 0.1 nM–10 μM) were performed. Basal NO synthase reactivity was quantified through the addition of L-NAME (200 μM) to aortic rings sub-maximally contracted with U46619 to 20–30% of maximal U46619 contraction.

### Histopathology

Aortic sections were fixed in 10% neutral buffered formalin, embedded in paraffin wax and cut into 4 µm sections. Vessels were stained with Masson’s trichrome for histological analysis of collagen and adipocytes. Each whole slide was scanned using the Aperio Scanscope AT Turbo, and raw virtual slide files were viewed using Aperio ImageScope software (Leica Biosystems Microsystems, Mount Waverley, VIC, Australia) to extract the whole image. Collagen content and adipocyte cross-sectional area was quantified by a semi-automated macro in ImageJ (version 1.53t, National Institute of Health, USA).

### Reagents

Drugs for wire myography were purchased from Sigma-Aldrich (St Louis, MO, USA), except for U46619 (Cayman Chemical, Ann Arbor, MI, USA). All drugs were dissolved in distilled water, except for indomethacin, which was dissolved in 0.1 mol/l sodium carbonate, and U46619, which was dissolved in 100% ethanol to create a stock solution at 1 mmol/l. All antibodies for flow cytometry were purchased from Biolegend, USA.

### Statistical analysis

Data are expressed as mean ± SEM with n representing the number of mice per group. All data analysis was performed in GraphPad Prism (version 7.0, GraphPad Software, San Diego, CA, USA. Concentration–response curves from aorta were fit to a sigmoidal curve using nonlinear regression. ACh- and SNP-evoked relaxation with and without blockers was expressed as a percentage of pre-contraction evoked by U46619. Body weight, fasting blood glucose, plasma insulin, blood pressure and concentration–response curve data were analyzed using a two-way ANOVA, with Sidak’s post hoc. Blood cholesterol, HbA1C, flow cytometry data, pEC50 and R_max_ data were compared using a two-way ANOVA, with Tukey’s *post-hoc* analysis. A level of *P* < 0.05 was considered significant. Power calculations indicated a minimum sample size of *n* = *7* for metabolic parameters, *n* = *5* for flow cytometry and *n* = *6* for wire myography was required to identify a 25% mean effect change in with 80% power, and standard deviation of 10%. Assessors and data collectors were blinded to experimental groups.

## Results

### HFD increased weight gain in both sexes, with sex-specific effects on metabolic parameters

**Figure 1:**
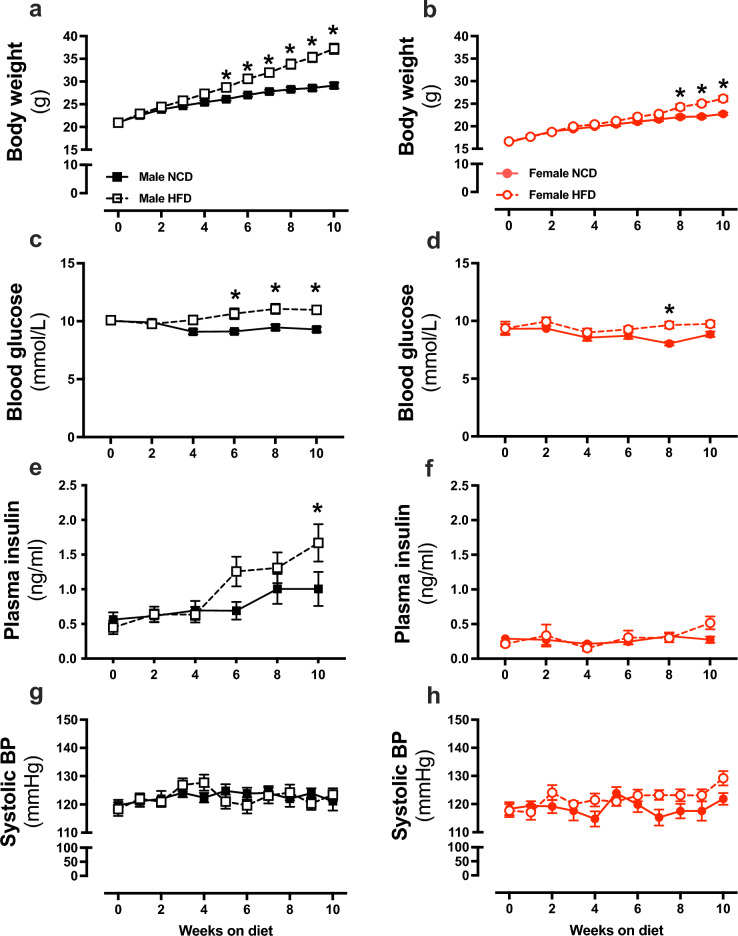
High fat diet-induced metabolic disturbances are delayed in female mice. Body weight **(a**,**b**), fasting blood glucose (**c**,**d**), fasting plasma insulin (**e**,**f**) and systolic blood pressure (**g**,**h**) from C57BL/6 male (black) and female (red) mice fed a normal chow diet (NCD; filled circle & filled square) or high-fat diet (HFD; open circle & open square). Values are mean ± SEM.* P* < 0.05 for 2-way ANOVA with Sidak’s multiple comparison. *NCD vs. HFD**,** (**a**–**d)** n = 10–17 per group, (**e**,**f**) n = 7–9 per group, (**g**,**h**) n = 10–12 per group.

HFD increased body weight in both males and females compared to NCD mice (*P* < 0.05; Fig. 1a,b). *Post hoc* analyses revealed that in males, body weight was increased from week 5 (*P* < 0.05; Fig. 1a). Interestingly, the onset of obesity was delayed in females where HFD increased bodyweight in only weeks 9 and 10 of the diet regimen (*P* < 0.05; Fig. 1b). HFD induced hyperglycemia in both sexes, but at different timepoints. HFD increased fasting blood glucose in males (*P* = 0.001) from week 6 of the diet regiment (P < 0.05; Fig. 1c). In females however, HFD increased fasting blood glucose transiently at week 8 (*P* = 0.001; Fig. 1d). HFD increased fasting plasma insulin at week 10 in males but did not change in females (Fig. 1e,f) and SBP remained unchanged in both sexes (Fig. 1g,h). At the endpoint, HFD increased blood cholesterol in males (*P* = 0.03) but not females (Tables 1, 2). HFD increased plasma estradiol in males (*P* = 0.02) but not females (Tables 1, 2). HFD did not affect blood triglycerides or HbA1c in either sex when compared to NCD control mice (Tables 1, 2).

**Table 1:** Blood cholesterol, triglycerides, glycated hemoglobin (HbA1c), and plasma estradiol in normal chow diet (NCD) and high-fat diet (HFD)-fed male mice.

	Males	
	NCD (n = 8–13)	HFD (n = 9–14)
Cholesterol (mg/dL)	111.3 ± 11.9	160.2 ± 14.6*
Triglycerides (mg/dL)	179.5 ± 14.9	191.5 ± 15.9
HbA1c (%)	4.5 ± 0.1	4.7 ± 0.1
Plasma estradiol (pg/mL)	3.7 ± 0.4	5.3 ± 0.5*

Data are mean ± SEM, *P < 0.05 vs NCD, Student unpaired *t* test.

**Table 2:** Blood cholesterol, triglycerides, glycated hemoglobin (HbA1c) and plasma estradiol in normal chow diet (NCD) and high-fat diet (HFD)-fed female mice.

	Females	
	NCD (n = 8)	HFD (n = 8–9)
Cholesterol (mg/dL)	83.4 ± 10.4	93.9 ± 10.5
Triglycerides (mg/dL)	118.3 ± 15.2	143.4 ± 19.4
HbA1c (%)	4.5 ± 0.1	4.5 ± 0.01
Plasma estradiol (pg/mL)	4.5 ± 0.7	5.5 ± 0.5

Data are mean ± SEM.

### Reduced aortic inflammatory monocytes in HFD-fed females but not HFD-fed males

**Figure 2:**
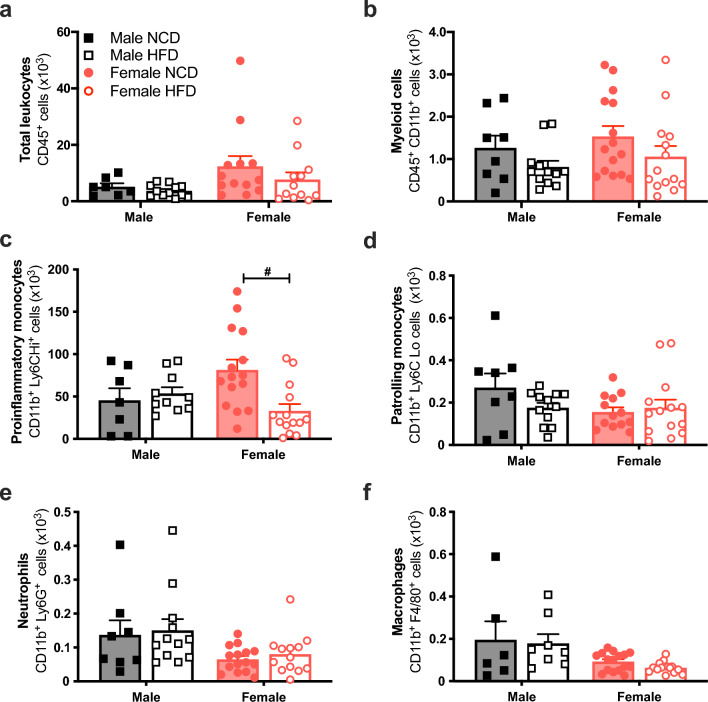
High fat diet reduces aortic proinflammatory monocytes in female mice. Total leukocytes (CD45^+^; (**a**)), myeloid-derived cells (CD11b^**+**^; (**b**)) proinflammatory monocytes (Ly6C^Hi^; (**c**)), patrolling monocytes (Ly6C^Lo^; (**d**)), neutrophils (Ly6G^+^; (**e**)), macrophages (F4/80^+^; (**f**)) in the aortas from C57BL/6 male (n = 5–8 per group; black) and female (n = 14–16 per group; red) mice fed a normal chow diet (NCD; filled circle & filled square ) or high-fat diet (HFD; open circle & open square). Values are mean ± SEM. *P* < 0.05 *Male NCD vs. Male HFD; ^#^Female NCD vs. Female HFD for 2-way ANOVA with Tukey’s multiple comparison.

**Figure 3:**
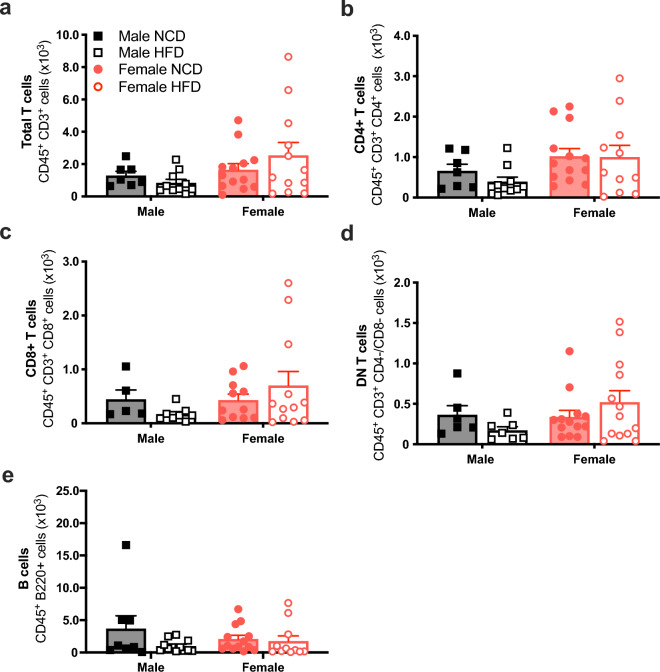
High fat diet did not affect aortic lymphocyte populations. Total T cells (CD3^+^; (**a**)), T helper/Treg cells (CD4^+^; (**b**)), cytotoxic T cells (CD8^+^; (**c**)), double-negative T cells (CD4^–^CD8^–^; (**d**)) and B cells (B220^+^; (**e**)) in the aortas from C57BL/6 male (black) and female (red) mice fed a normal chow diet (NCD; filled circle & filled square) or high-fat diet (HFD; open circle & open square). Values are mean ± SEM, n = 5–13 per group. *P* < 0.05 *Male NCD vs. Male HFD; ^#^Female NCD vs. Female HFD for 2-way ANOVA with Tukey’s multiple comparison.

Flow cytometry revealed that there was a sex effect independent of HFD for the total number of infiltrated leukocytes (CD45^+^, Fig. 2a, P = 0.046), macrophages (CD11b^+^F4/80^+^, Fig. 2f, P = 0.001) and CD4^+^ T cells (CD45^+^CD3^+^CD4^+^, Fig. 3b, P = 0.03). HFD had no effect on the total number of infiltrated leukocytes (CD45^+^) or myeloid-derived cells (CD45^+^CD11b^+^) in the aorta in either sex (Fig. 2a,b). However, HFD decreased aortic proinflammatory monocytes (CD11b^+^Ly6C^Hi+^; *P* = 0.005; Fig. 2c) in females but not in males. Further analysis of other myeloid-derived cell types revealed that HFD did not affect patrolling monocytes (CD11b^+^Ly6C^Lo+^), neutrophils (CD11b^+^Ly6G^+^) or macrophages (CD11b^+^F4/80^+^) in either sex (Fig. 2df). Moreover, HFD did not alter any of the aortic lymphocyte populations studied. This included total T cells (CD45^+^CD3^+^, Fig. 3a), CD4^+^ T cells (CD45^+^CD3^+^CD4^+^, Fig. 3b), CD8^+^ T cells (CD45^+^CD3^+^CD8^+^, Fig. 3c), other double-negative T cells (CD45^+^CD3^+^CD4^-^CD8^-^, Fig. 3d) and B cells (CD45^+^B220, Fig. 3e).

### HFD-feeding impairs maximal endothelium-dependent vasorelaxation in males but not females

**Figure 4:**
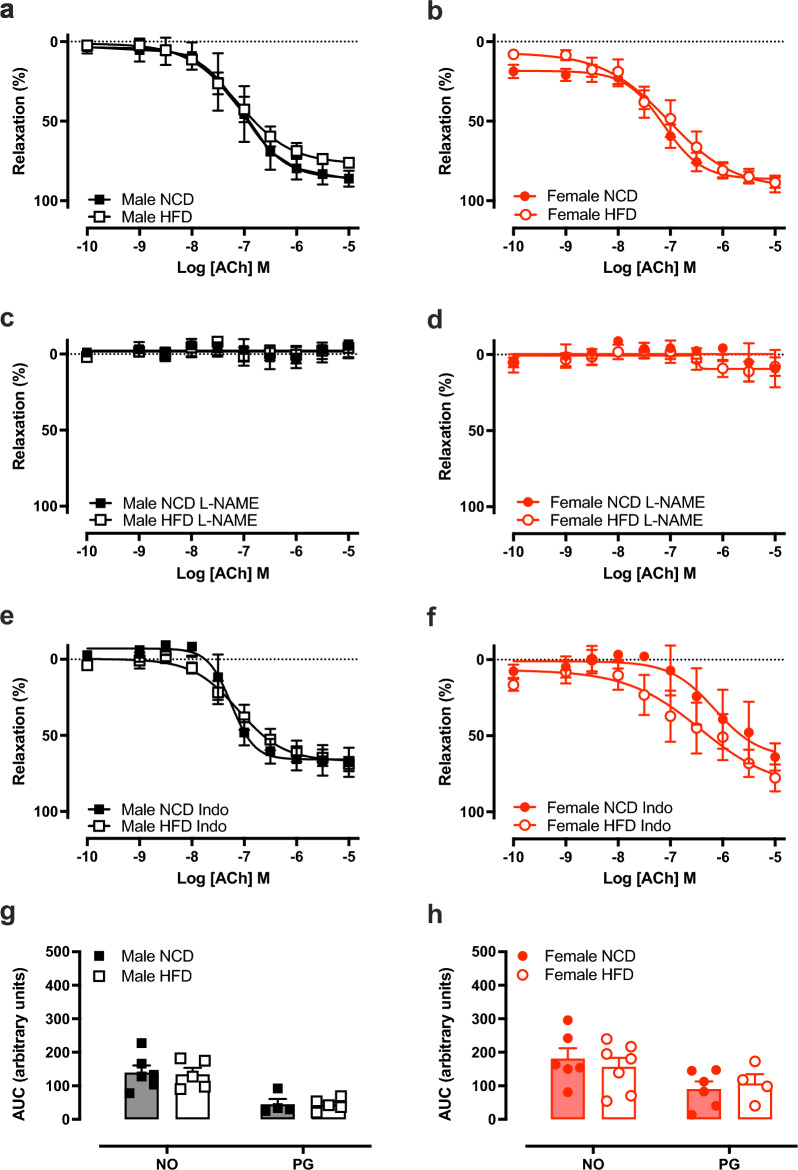
High fat diet impaired endothelium-dependent relaxation in male mice. Concentration–response curves to the acetylcholine (ACh) in the absence (**a**,**b**) and presence of L-NAME (**c**,**d**) or indomethacin (Indo; (**e**,**f**)) and area under the curve analyses (**g**,**h**) in C57BL/6 male (black) and female (red) mice fed a normal chow diet (NCD; filled circle & filled square) or high-fat diet (HFD; open circle & open square). *NO* nitric oxide, *PG* prostanoids. Values are mean ± SEM, n = 6–7 per group. *P < 0.05 vs. NCD, for 2-way ANOVA with Bonferroni’s multiple comparison (**a**–**f**) or mixed-effects model with Sidak’s multiple comparison (**g**,**h**).

**Figure 5:**
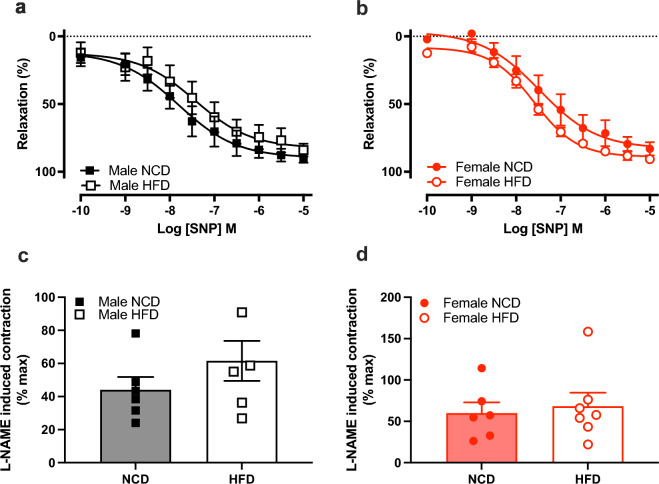
High fat diet did not alter relaxation to sodium nitroprusside or basal NO. Male and female concentration–response curves to sodium nitroprusside (SNP; (**a**,**b**)) and maximal contraction to L-NAME (**c**,**d**) in C57BL/6 male (black) and female (red) mice fed a normal chow diet (NCD; filled circle & filled square) or high-fat diet (HFD; open circle & open square). Values are mean ± SEM, n = 6–7 per group. *P < 0.05 vs. NCD, 2-way ANOVA with Bonferroni’s multiple comparison (**a**,**b**) or Student unpaired *t* test (**c**,**d**).

There were no differences in ACh concentration–response curves between diet groups in male or female mice (Fig. 4a,b, Tables 3, 4). However, there was an impaired maximal relaxation in males on HFD versus NCD (*P* = 0.003), but no diet effect in females (Tables 3, 4). To investigate the effect of obesity on the mechanisms of endothelium-dependent relaxation, concentration–response curves to ACh were evaluated in the presence of the NOS inhibitor, L-NAME or the COX inhibitor, indomethacin. There were no differences in ACh concentration–response curves between diet groups in the presence of either blocker (Fig. 4c–f). Area under the curve was calculated to determine contribution of NO and COX. HFD did not change the contributions of either mechanism to ACh-mediated relaxation of either sex (Fig. 4g,h). Similarly, HFD did not affect endothelium-independent relaxation to the NO donor SNP, L-NAME-induced contraction, or half-maximal effective concentration to ACh (Fig. 5a–d, Tables 3, 4).

**Table 3:** The half-maximal effective concentration (pEC_50_) and maximal relaxation (R_max_) for acetylcholine (ACh) and sodium nitroprusside (SNP) in the aorta from male mice fed a normal control diet (NCD) or high-fat diet (HFD).

Conditions		NCD		HFD	
		pEC_50_	R_max_ (%)	pEC_50_	R_max_ (%)
Male	ACh alone	7.1 ± 0.1	86.1 ± 1.8	7.1 ± 0.2	76.6 ± 1.9*
	ACh + L-NAME	7.4 ± 0.7	− 6.4 ± 9.2	8.9 ± 0.5	− 7.2 ± 5.7
	ACh + Indo	7.1 ± 0.1	67.7 ± 9.6	7.2 ± 0.2	68.8 ± 4.5
	SNP	7.8 ± 0.3	90.2 ± 3.0	7.7 ± 1.9	84.0 ± 4.6

ACh concentration–response curves were performed in the absence or presence of L-NAME or indomethacin (Indo). Data are expressed as mean ± SEM, *P < 0.05 vs NCD.

**Table 4:** The half-maximal effective concentration (pEC_50_) and maximal relaxation (R_max_) for acetylcholine (ACh) and sodium nitroprusside (SNP) in the aorta from female mice fed a normal control diet (NCD) or high-fat diet (HFD).

Conditions		NCD		HFD	
		pEC_50_	R_max_ (%)	pEC_50_	R_max_ (%)
Female	ACh alone	6.9 ± 0.2	89.5 ± 5.3	7.2 ± 0.3	88.5 ± 3 0.1
	ACh + L-NAME	2.2 ± 2.6	9.2 ± 12.1	2.9 ± 1.4	7.9 ± 5.9
	ACh + Indo	6.4 ± 0.2	63.9 ± 8.9	7.2 ± 0.5	77.7 ± 8.7
	SNP	7.7 ± 0.3	83.1 ± 4.8	7.6 ± 0.1	90.7 ± 2.8

ACh concentration–response curves were performed in the absence or presence of L-NAME or indomethacin (Indo). Data are expressed as mean ± SEM.

### HFD increased adipocyte size in males with no changes in collagen

**Figure 6:**
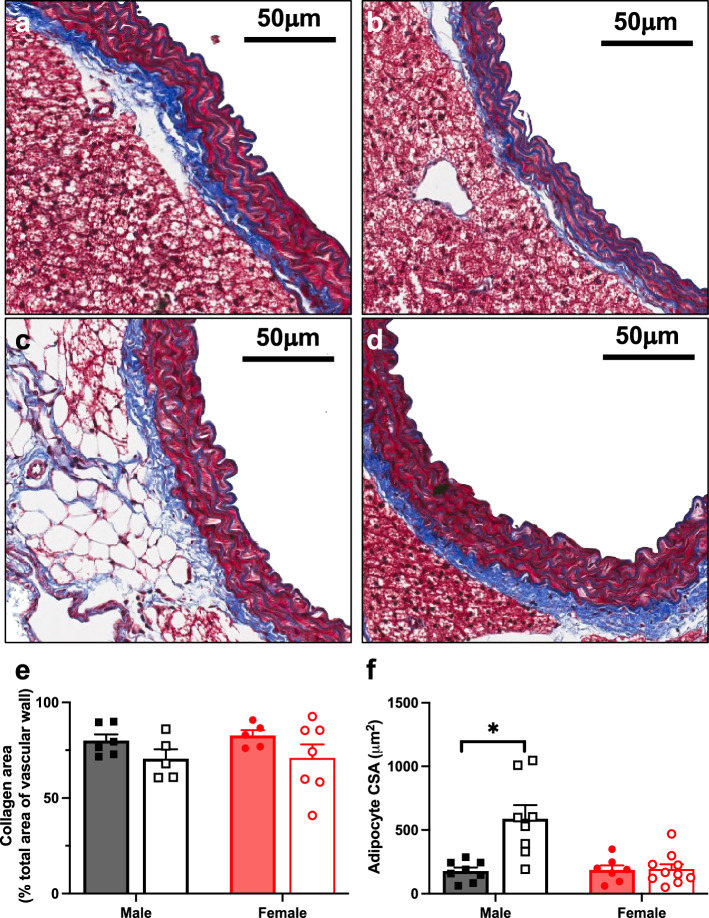
High fat diet increased aortic perivascular adipocyte size in male mice. Representative images of thoracic aorta stained with Masson’s trichrome from (**a**) NCD male, (**b**) NCD female, (**c**) HFD male and (**d**) HFD female. Images were scanned at 10 × magnification. Collagen area (as a percentage of the total area of the vascular wall; (**e**) and average adipocyte cross sectional area (CSA; (**f**) in C57BL/6 male (black) and female (red) mice fed a NCD (filled circle & filled square) or HFD (open circle & open square; (**e**). Scale bar = 50 μm. Values are mean ± SEM, n = 5–10 per group. *P < 0.05 vs. NCD, for 2-way ANOVA with Sidak’s multiple comparison test.

To assess the effect of HFD on aortic collagen content and perivascular adipocyte size, we performed Masson’s trichrome staining. Aortic collagen content remained unchanged in response to HFD for both sexes (Fig. 6a–e), but HFD increased the mean area of aortic adipocytes in males (*P* = 0.003), but not females (Fig. 6a,c,f).

## Discussion

Sexual dimorphisms in diet-induced models of obesity are understudied, with a bias towards studies in males. Our study addressed this gap by assessing vascular inflammation and function in male and female mice in response to the HFD. We showed that a 10-week HFD regimen induced obesity (increased body weight), hyperglycemia, hyperinsulinemia, and hypercholesterolemia in males. The development of these metabolic disturbances was blunted or absent in females. We also showed that HFD caused mild endothelial dysfunction in the aorta, but only in males. Conversely, HFD only altered aortic leukocytes in females – specifically, it resulted in fewer proinflammatory monocytes.

Our finding that males are more susceptible to HFD-induced metabolic disturbances than females is consistent with other studies that reported earlier HFD-induced weight gain in males than females^15,36,37^. Aligned with our findings, increased weight gain in males is evident after 5 weeks of HFD, but not until later in females^13^. While most studies have reported that excessive weight gain is blunted or delayed in females, one study reported similar weight gain between sexes after 14 weeks of HFD^38^. This difference could be attributed to the HFD being introduced to the mice at a later age (i.e. 11–12 weeks of age) than most other studies^38^. Total cholesterol was increased in HFD males, with no changes in total triglycerides. This contrasts with females, where HFD had no effect on cholesterol or triglycerides. In other models of advanced HFD-induced obesity (i.e. 12–32 weeks of HFD), plasma lipids and cholesterol were increased in both sexes^39^. Taken with our data, it is likely that dyslipidemia is delayed in females, rather than being completely absent.

HFD males had higher fasting blood glucose levels from week 6 and higher fasting plasma insulin levels from week 10 than NCD males. In contrast, HFD females only had a transiently higher fasting blood glucose at week 8, with no change in fasting plasma insulin. Consistent with other studies^15,36,38^, this suggests that insulin resistance occurs within 10 weeks of HFD in males, but not in females. The sexual dimorphism in insulin resistance is well-established, with males exhibiting a greater degree of insulin resistance compared to females. This disparity could be due to sex-specific traits, such as elevated visceral and hepatic adipose tissue, coupled with the absence of protective estrogen and lower adiponectin (an insulin-sensitizing hormone levels^40^. Despite insulin resistance occurring in males (and transiently in females), HFD did not affect glycated hemoglobin (HbA1c). This was expected, as blood glucose had been elevated for only a short period. HbA1c is an indicator of chronically increased blood glucose levels (i.e. ≥ 3 months). Indeed, studies using longer HFD regimens (16–24 weeks) did report increases in both fasting blood glucose and HbA1c^41–43^.

HFD increased plasma estradiol in males but not females. This was particularly interesting given the established protective effects of estrogen against obesity and metabolic complications in male mice^44^. Previous studies have reported that administration of estrogen in obese male mice was associated with improved weight gain and diet-induced metabolic changes^44^. However, the observed increase in estradiol in response to HFD is an endogenous response and corroborates with results in human males that also show elevated circulating estradiol in response to diets high in polyunsaturated fats whereby increased aromatase activity converts testosterone to estradiol^45,46^. Furthermore, the Barostoc feed used for NCD may contain isoflavones, a natural estrogenic compound found in legumes^47^. Consequently, the NCD groups may exhibit higher levels of plasma estradiol, which may mask a greater difference in plasma estradiol between NCD and HFD groups. One limitation of our study was that we did not account for the estrus cycle in female mice. There are studies that suggest that the rodent estrus cycle can have an impact on blood pressure, blood glucose, and estradiol levels^48,49^. By not including this, we may be potentially masking important differences between our experimental groups. Furthermore, we are also aware of the challenges associated with measuring plasma estradiol levels using direct immunoassays, especially at low concentrations^50,51^. However, the assays used were sensitive enough to detect estradiol levels in our study, and no significant differences in estradiol levels were observed between the groups.

Our flow cytometry data showed no effect of HFD on aortic immune cells in either sex. Surprisingly, we observed fewer Ly6CHi^+^ proinflammatory monocytes in females on HFD compared to NCD. This suggests a female-specific compensatory mechanism to suppress aortic macrophages in response to HFD. This contrasts to other rodent HFD studies where HFD-induced obesity increased aortic inflammation in males – characterized by macrophage infiltration and secretion of inflammatory cytokines (interleukin-6, interleukin-8, tumor necrosis factor-α and monocyte chemoattractant-1)^52–54^. However, it is important to note that no previous studies included females and had a diet regimen longer than 10 weeks.

Aortic lymphocytes (T and B cells) remained unchanged in response to HFD. There were also no sex-specific responses in aortic lymphocytes. This was surprising as obesity-related chronic inflammation is typically associated with increased lymphocyte infiltration within adipose tissues^53^. However, previous HFD studies characterized T and B cells in visceral adipose tissues rather than the aorta (or aortic perivascular adipose tissue) and used longer diet regimens. Other HFD studies show increased CD8^+^ T cells and decreased CD4 + and regulatory T cells in the visceral adipose of obese males when compared to HFD females^52,54^. Moreover, Treg populations are typically reduced in visceral adipose tissue in mice fed a HFD^55,56^. To the best of our knowledge, no previous studies have characterized leukocytes in aortic perivascular adipose tissue in obese rodents. While our findings suggest that the typical changes to T cells do not occur in aortic perivascular adipose tissue in early obesity, more research is required to confirm this in models of advanced obesity.

Despite a slight reduction in the maximal vasorelaxation to ACh in HFD males, endothelial function was similar between HFD and NCD mice. This contrasts with other studies where HFD induced substantial endothelial dysfunction after ≥ 12 weeks^18,57,58^. Our 10-week HFD regimen was possibly not long enough to significantly reduce endothelial vasorelaxant function. Interestingly, one study reported that 8 weeks on a HFD increased aortic inducible NOS (iNOS) expression and NO production in male mice^59^. This suggests a short-term compensatory mechanism stimulated by inflammatory cytokines, however prolonged iNOS-derived NO can promote endothelial dysfunction^59^. In our study of early obesity, males were more vulnerable to metabolic disturbances compared to HFD-females. However, previous studies in rodents on western diet for 16 weeks demonstrated sexual dimorphism in obesity-related endothelial stiffening^60^. This showed that females exhibit greater susceptibility to endothelial stiffening due to estrogen signaling when compared to males. This is observed in a clinical setting where diabetic women lose the cardiovascular protection of estrogen and consequently, exhibit higher incidence and severity of cardiovascular disease compared to men^60^. Therefore, our wire myography data, when combined with the observed metabolic disturbances, suggests that hyperglycaemia, hyperinsulinemia, and weight gain can precede endothelial dysfunction in the aorta in HFD-fed male mice.

Endothelium-mediated relaxation in response to ACh when pharmacologically blocked with L-NAME or indomethacin was comparable between diet groups in both sexes. Therefore, we were unable to determine whether the mild impairment in maximal relaxation to ACh in HFD males was due to NO or prostanoids. Our AUC analyses revealed that aortic ACh-mediated relaxation predominantly occurred via NO, meaning it is more likely that impaired relaxation was due to a reduction of the NO contribution. Notably, prostanoids contribute significantly to ACh-induced relaxation in human saphenous veins and internal mammary arteries^61^. However, the contribution of prostanoids to ACh-induced relaxation in the human aorta has not been explored, therefore it is difficult to compare our findings to that in humans. Due to limited tissue availability, we were unable to further interrogate NO or prostanoid pathways.

Lastly, our data show that HFD resulted in larger aortic perivascular adipocytes in males but not females. While adipose tissue expansion is a well-recognized driver of inflammation, we did not observe aortic inflammation despite the increase in adipocyte size^62^. However, HFD males did exhibit hyperglycemia and hyperinsulinemia, consistent with adipocyte expansion accompanying metabolic disturbances but preceding inflammation. Moreover, unchanged adipocyte size coupled with fewer proinflammatory monocytes in HFD females suggests a protective mechanism exists within the perivascular adipose tissue of females, which may contribute to preserved endothelial function; however, this is a limitation of our study. Although perivascular adipose tissue was included in the flow cytometry studies, it was not included in the wire myography studies. Consequently, we were unable to examine the impact of HFD on perivascular adipose tissue and its secretory factors that could influence vascular function^63^. Additionally, further investigations characterizing aortic function, adipocyte size and inflammation and at different time points during a longer HFD diet study are required to clearly define the sequence of events occurring within the aorta.

While the primary objective of this study was to explore sex-specific responses to the HFD, the significance of understanding sex differences is also acknowledged. Unfortunately, the study was not powered to perform 3-way ANOVAs to investigate sex differences. This is a limitation of the study. Future studies should aim to focus on sex differences to gain a comprehensive understanding of the underlying biological mechanisms that contribute to the variation between males and females.

Our study has characterized, for the first time, the early sex-specific effects of a HFD on metabolic parameters, vascular inflammation and vasorelaxant function in male and female mice. We identified that mild vascular dysfunction is a male-specific effect of HFD – likely influenced by the increased vulnerability to metabolic disturbances. Indeed, metabolic disturbances were blunted in HFD females and vascular function was preserved. Moreover, HFD reduced proinflammatory monocytes in females, which may have contributed to the protection from vascular dysfunction. Overall, our study highlights that sex-specific effects to HFD are apparent in the aorta during the early stages of obesity or pre-obesity, but further work is still needed to understand the implications of these differences in the later stages of disease.

### Supplementary Information

Supplementary Information.

## Data Availability

The datasets generated and/or analyzed during the current study are available from the corresponding author on reasonable request.
