# Supplementary material for: Sex-specific effects of a high fat diet on aortic inflammation and dysfunction
Source: Sci Rep. 2023 Dec 8;13:21644. doi: 10.1038/s41598-023-47903-1 (PMC10703842; doi:10.1038/s41598-023-47903-1)
Supplement: Supplementary file 1 — Supplementary Information. [file 41598_2023_47903_MOESM1_ESM.docx]

**Sex-specific effects of a high fat diet on aortic inflammation and dysfunction.**

Vivian Tran^1^, Holly Brettle^1^, Henry Diep^1^, Quynh Nhu Dinh^1^, Maeve O’Keeffe^1^, Kerry V Fanson^2^, Christopher G Sobey^1^, Kyungjoon Lim^1^, Grant R Drummond ^1^, Antony Vinh^1*^, Maria Jelinic^1*^

^1^Centre for Cardiovascular Biology and Disease Research, Department of Microbiology, Anatomy Physiology and Pharmacology, School of Agriculture, Biomedicine and Environment, La Trobe University, Bundoora, VIC, Australia, ^2^ Department of Animal, Plant and Soil Sciences, School of Agriculture, Biomedicine and Environment, La Trobe University, Bundoora, VIC, Australia.

* Equal contribution

**Running Head (60 characters max):** Sex-specific effects of obesity in the aorta

**Corresponding author:** Dr Maria Jelinic

Centre for Cardiovascular Biology and Disease Research,

La Trobe University,

Bundoora, VIC,

Australia

Ph.: +61 3 9479 3631

Fax: +61 3 9479 3660

[m.jelinic@latrobe.edu.au](mailto:m.jelinic@latrobe.edu.au)

**Co-corresponding author:** A/Prof Antony Vinh

[a.vinh@latrobe.edu.au](mailto:a.vinh@latrobe.edu.au)

**Suppl Table 1.** Antibodies used for flow cytometry. All antibodies were purchased from Biolegend (San Diego, California, USA)

| **Antibody** | **Clone** | **Fluorophore** | **Concentration** |
| --- | --- | --- | --- |
| CD45 | 30-F11 | AF700 | 1 μg/ml |
| CD11b | M1/70 | BV421 | 0.5 μg/ml |
| Ly6C | HK1.4 | FITC | 0.2 μg/ml |
| Ly6G | 1A8 | PE-Cy7 | 0.2 μg/ml |
| F4/80 | BM8 | APC-Cy7 | 0.4 μg/ml |
| CD3e | 17A2 | APC | 0.4 μg/ml |
| CD4 | RM4-5 | BV605 | 0.4 μg/ml |
| CD8a | 53-6.7 | PerCP Cy5.5 | 0.2 μg/ml |
| B220 | RA3-6B2 | PE | 0.2 μg/ml |


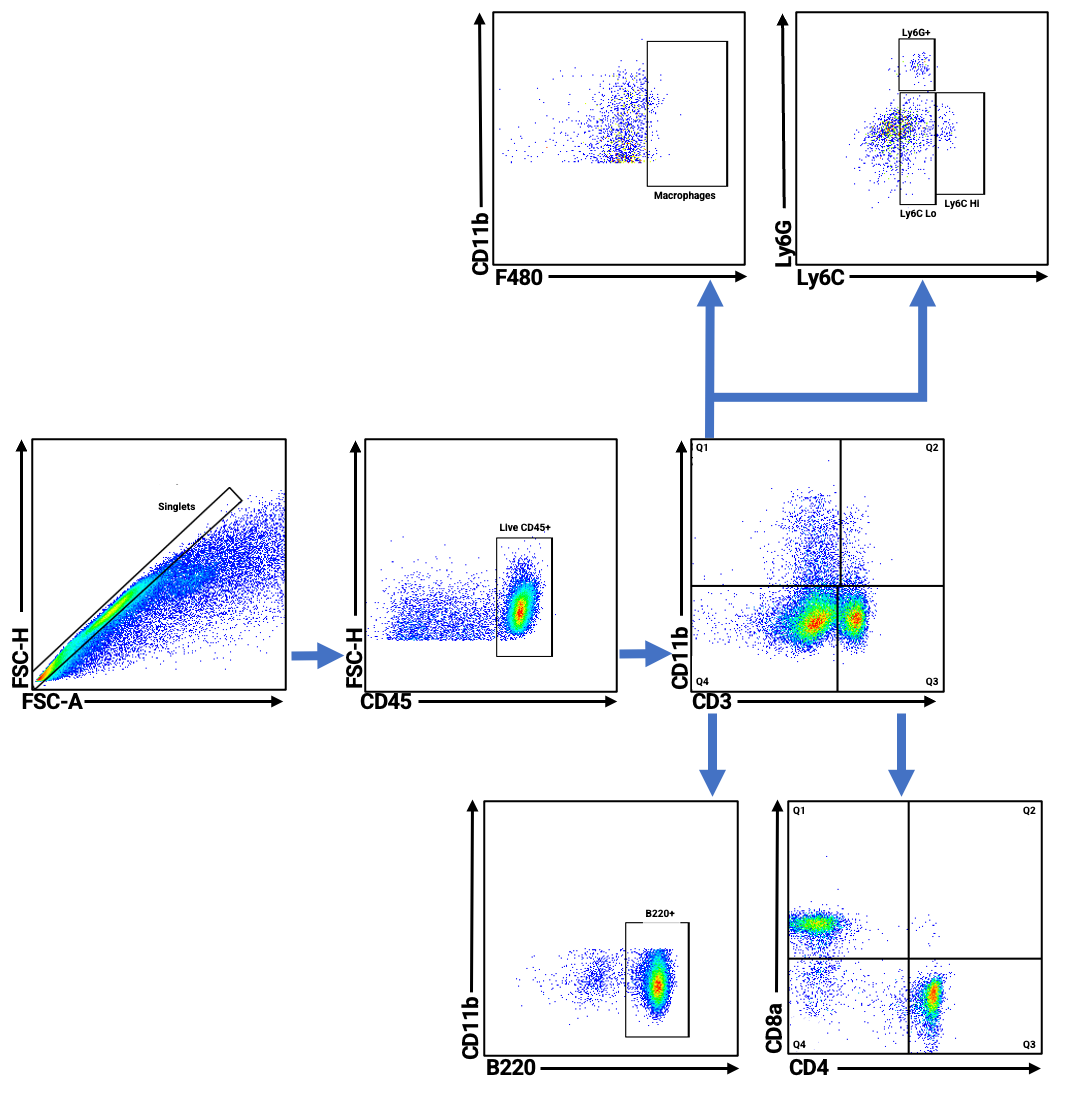


**Suppl Figure 1.** Gating strategy for flow cytometric analysis. Leukocytes were gated as CD45+ populations against forward scatter (FSC). Total leukocytes, i.e. live leukocyte singlets, were gated by FSC -height vs FSC-area, and exclusion of dead cells (live/dead stain). Total leukocytes were then divided into myeloid cells (CD11b+) and T cells (CD3+). Some of the myeloid cells were identified as macrophages (CD11b+F4/80+), proinflammatory monocytes (Ly6CHi), patrolling monocytes (Ly6CLo) and neutrophils (Ly6G+). B cells were positive for B220+. T cells that were positive for CD3 staining were further classified as CD4+, or CD8+ T cells.
